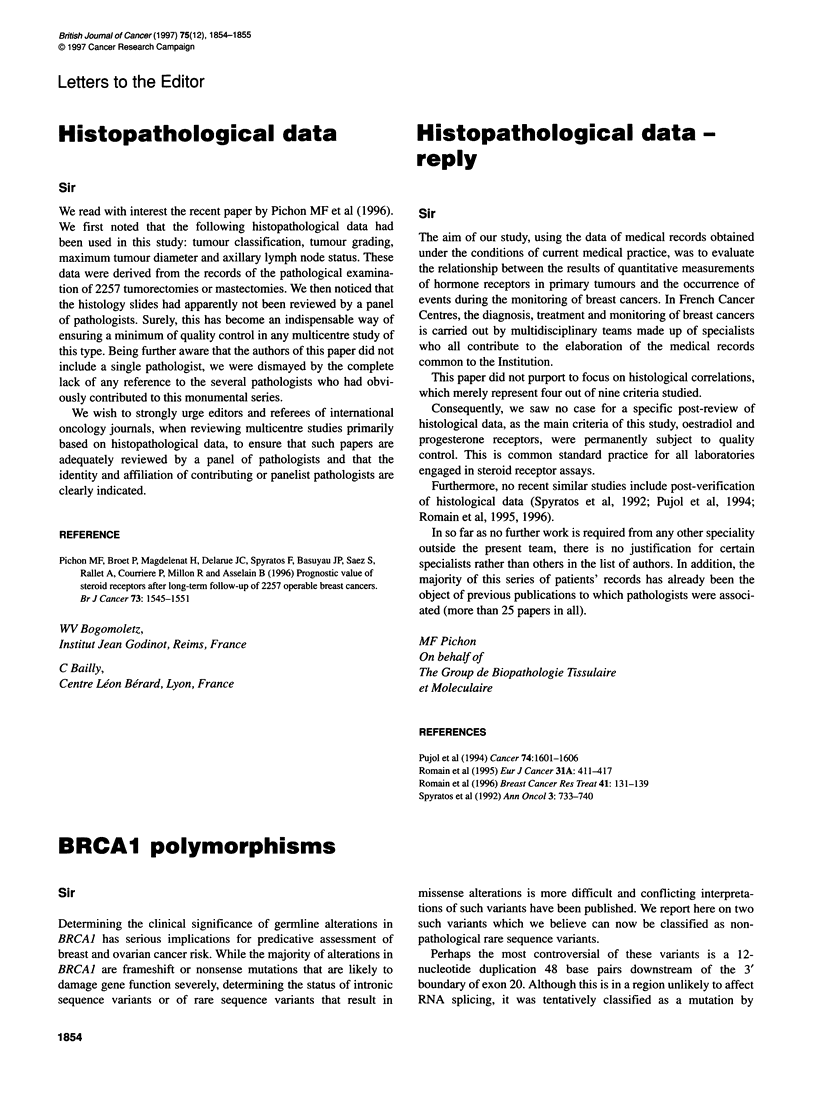# Histopathological data.

**DOI:** 10.1038/bjc.1997.316

**Published:** 1997

**Authors:** W. V. Bogomoletz, C. Bailly


British Journal of Cancer (1997) 75(12), 1854–1855
© 1997 Cancer Research Campaign

Letters to the Editor

### Histopathological data

**Sir**

We read with interest the recent paper by Pichon MF et al (1996). We first noted that the following histopathological data had been used in this study: tumour classification, tumour grading, maximum tumour diameter and axillary lymph node status. These data were derived from the records of the pathological examination of 2257 tumorectomies or mastectomies. We then noticed that the histology slides had apparently not been reviewed by a panel of pathologists. Surely, this has become an indispensable way of ensuring a minimum of quality control in any multicentre study of this type. Being further aware that the authors of this paper did not include a single pathologist, we were dismayed by the complete lack of any reference to the several pathologists who had obviously contributed to this monumental series.

We wish to strongly urge editors and referees of international oncology journals, when reviewing multicentre studies primarily based on histopathological data, to ensure that such papers are adequately reviewed by a panel of pathologists and that the identity and affiliation of contributing or panelist pathologists are clearly indicated.

### REFERENCE

Pichon MF, Broet P, Magdelenat H, Delarue JC, Spyratos F, Basuyau JP, Saez S, Rallet A, Courriere P, Millon R and Asselain B (1996) Prognostic value of steroid receptors after long-term follow-up of 2257 operable breast cancers. *Br J Cancer* **73**: 1545–1551

*WV Bogomoletz,*
*Institut Jean Godinot, Reims, France*

*C Bailly,*

*Centre Léon Bérard, Lyon, France*